# Role of human Myocyte Enhancer Factor 2 (MEF2) proteins in cancer: structural insights, functional diversity, and regulatory mechanisms

**DOI:** 10.1186/s12935-025-03995-5

**Published:** 2025-10-15

**Authors:** Ayush Khandelwal, Amir H. Fathi, Sachin Shetty, Arijit Mukhopadhyay, Vasudha Devi, Shama Prasada Kabekkodu

**Affiliations:** 1 Department of Cell and Molecular Biology, Manipal School of Life Sciences, Manipal Academy of Higher Education, Manipal, 576104 Karnataka India; 2 Biomedical Research and Innovation Centre, School of Science, Engineering & Environment, University of Salford Manchester, Salford, M5 4WT UK; 3 Department of Basic Medical Sciences, Manipal Academy of Higher Education, Manipal, 576104 Karnataka India

**Keywords:** MEF2 (Myocyte enhancer factor 2) proteins, Transcription factor, Epigenetic regulation, Cell differentiation, Apoptosis, Stress response, Cancer signalling

## Abstract

The Myocyte Enhancer Factor 2 (MEF2) family of transcription factors plays a central role in regulating key cellular processes, including differentiation, proliferation, apoptosis and stress response. MEF2 activity is important for normal physiological development and tissue homeostasis, and its dysregulation has been implicated in a wide spectrum of disorders, including developmental anomalies, metabolic syndrome, neurodegenerative disease, and various cancers. The functional adaptability of MEF2 arise from its dynamic regulation by histone deacetylases (HDACs) and kinase such as calcium/calmodulin-dependent protein kinase (CaMK) and mitogen-activated protein kinase (MAPK), allowing it to act as both a transcriptional activator and repressor. Beyond its transcriptional roles, MEF2 influences inflammation, cell cycle control, and adaptive responses to cellular stress, highlighting its importance in health and disease. Despite substantial progress, the molecular mechanism of MEF2 regulation and function remains poorly understood. This review systematically analyzed recent advances, critical knowledge gap, and proposes future directions for research in the area of MEF2. Understanding MEF2 signaling could reveal new therapeutic targets and enable precision treatments for related disorders.

**Graphical abstract:**

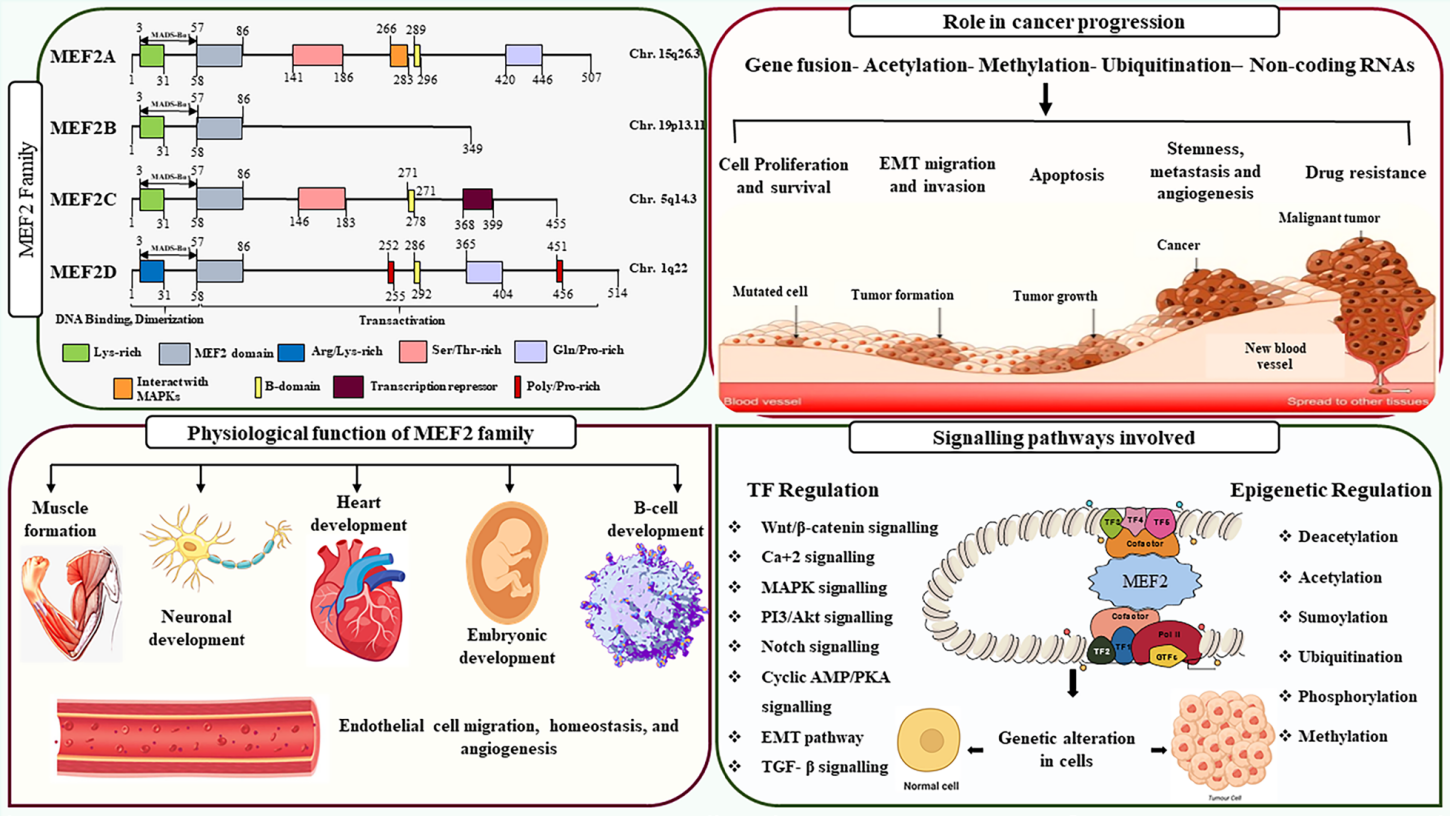

## Highlights

- MEF2 transcription factors play a central regulatory role in cancer hallmarks.
- Distinct functional domains of MEF2 (MADS-box, MEF2 domain, transcriptional activation/repression domains) and their distinctive roles.
- Context dependent function of isoforms of MEF2.
- Transcription regulation and post-translational modification of MEF2.
- Therapeutic potential of MEF2 family in cancer.

## Introduction

Transcription factors (TFs) are proteins that control gene expression by binding to specific DNA sequences and thereby regulating gene expression to control different cellular activities [1]. TF function is primarily mediated by the recruitment of diverse cofactors. While lacking intrinsic DNA-binding capability, these cofactors are precisely localized to genomic targets through specific protein-protein interactions with DNA-bound TFs, enabling the assembly of regulatory complexes [2]. Cofactors engage in a wide range of transcriptional modulation such as histone modification to chromatin remodeling, and direct interaction with the core transcriptional machinery [3]. TF-cofactors interaction is highly context-dependent as they vary based on cell types, extracellular or intracellular signals, and are often dysregulated during disease state. These interactions help cells to regulate complex gene expression programs required for normal homeostasis of the cell [4]. TFs generally function like molecular switches that control key cellular processes including differentiation, metabolism, proliferation and environmental adaptation [5]. Deregulated expressions of TFs and its target proteins have shown to play critical role in the development and progression of diseases. Thus, several studies are currently underway to target TF and their cofactors for improved clinical management of various diseases [1].

The Myocyte Enhancer Factor 2 (MEF2) is a TFs family consisting of four members (MEF2A, MEF2B, MEF2C, and MEF2D) that regulate diverse cellular processes like growth, differentiation and response to extracellular signals. MEF2 can either activate or repress gene transcription based on cell type and availability of interacting partners [6]. Cells such as cardiomyocytes show high levels of HDAC expression and low levels of MEF2 expression unless stimulated by signals such as calcium/calmodulin-dependent protein kinase (CaMK) and mitogen-activated protein kinase (MAPK). CaMK signalling has shown that it stimulates MEF2 by expelling histone deacetylases (HDAC4 and HDAC5), which would normally keep MEF2 in an inactive state [7]. MEF2 can also be stimulated by MAPK pathway via phosphorylating its transactivation domain (TAD) [8]. Both the CaMK and MAPK pathways are essential for MEF2 activation as they bind to distinct regions of the protein and work in coordination [9]. In contrast, MEF2 exhibits higher activity and showed lesser dependency from calcium signalling in cells that show lower HDAC expression, such as certain neurons or immune cells [10, 11].

The significance of MEF2 in brain development and its possible link to neuronal dysfunction is reported in many studies [12]. Interestingly, recent studies highlighted the contribution of MEF2 TFs in cerebellar dysfunction [13]. In the cerebellar cortex, MEF2A stimulates the formation of dendritic claws when sumoylated at Lys-403, but its acetylated form prevents postsynaptic granule neuron differentiation [14]. Previous studies have identified MEF2-regulated gene networks to govern multiple physiological processes, such as cardiomyocyte differentiation [15], skeletal muscle development [16], endothelial integrity [17], vascular homeostasis [17], B-cell differentiation [18], neurogenesis [19], cortical development [12], hematopoietic stem cell regulation [20], and apoptosis regulation [21]. These functional diversity and multiple regulatory role suggest the crucial role of MEF2 proteins in organs such as brain, heart, skeletal muscle, vasculature, and immune system [15].

**Fig. 1:**
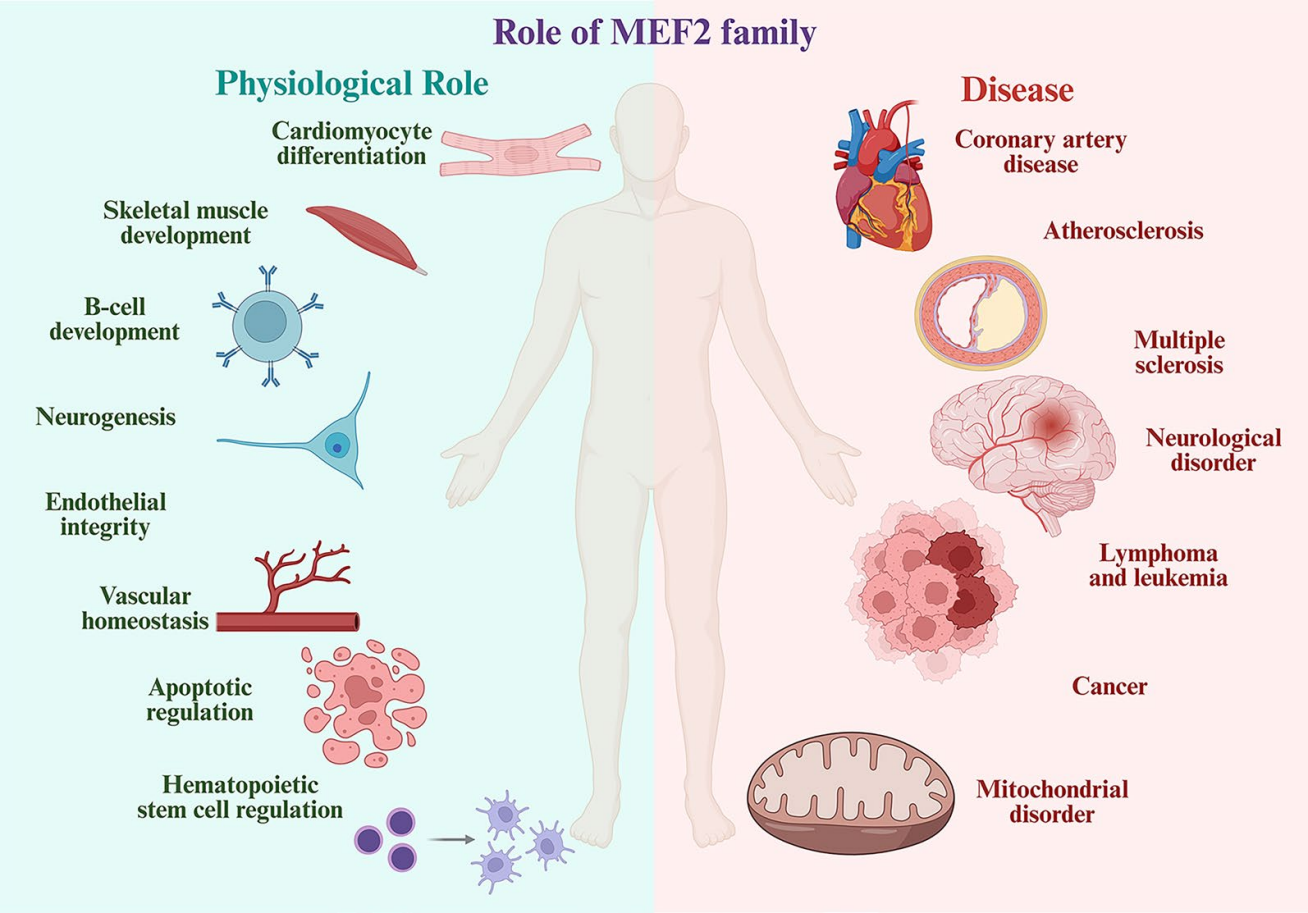
Physiological and Pathological Roles of MEF2 family: The MEF2 protein family plays diverse roles in normal physiology and disease. Under healthy conditions, MEF2 contributes to cardiomyocyte differentiation, skeletal muscle development, B-cell development, neurogenesis, endothelial integrity, vascular homeostasis, apoptotic regulation, and hematopoietic stem cell regulation. Dysregulation of MEF2 is linked to various diseases, including coronary artery disease, atherosclerosis, multiple sclerosis, neurological disorders, lymphoma and leukemia, cancer, and mitochondrial disorders (Created in BioRender. Kabekkodu, S. (2025) https://BioRender.com/5oa77tb)

Abnormal expression of MEF2 is connected with several diseases such as coronary artery disease and atherosclerosis [22], lymphoma and leukemia [23], Parkinson’s disease [24], Alzheimer’s disease [25], cortical malformations [26], multiple sclerosis [27], and mitochondrial disorders [28]. More recent studies have highlighted the role of aberrant MEF expression with cancer progression and metastasis [29]. These studies highlight the importance of MEF2 family of TFs in health and diseases (Fig. 1).

## MEF2 proteins: structure and their function

The MEF2 family of TFs is responsible for the regulation of cell growth and differentiation of genes in a wide range of organisms [30]. Initially recognized as a master regulator of skeletal muscle development, MEF2 proteins are discovered to bind A/T-rich DNA sequences in promoters of genes such as the muscle creatine kinase (MCK) to regulate muscle-specific gene expression [29]. Evolutionarily, the MEF2 gene family originated from an ancestral gene in lower eukaryotes like *Saccharomyces cerevisiae*, *Caenorhabditis elegans*, and *Drosophila melanogaster* (Table 1). These species have only one MEF2 homolog that can control essential process connected with development and differentiation processes, indicating a efficient transcriptional control mechanism [31]. Unlike simpler organisms, vertebrates possess four distinct MEF2 isoforms designated as MEF2A, MEF2B, MEF2C and MEF2D that regulates specialized and tissue-specific functions [32].

**Table 1:** Conservation of MEF2 genes across species

Species	Gene copy number	Approx. Sequence Identity to Human MEF2			Reference
		MADS domain	MEF2 domain	TAD domain	
*Homo sapiens*	4 (MEF2A, MEF2B, MEF2C, MEF2D)	100% 91% 98% 95%	100% 68% 87% 82%	100% 6% 11% 16%	[33]
*Drosophila melanogaster*	1 (dMEF2)	90%	68%	14%	[94]
*Caenorhabditis elegans*	1 (mef-2)	95%	84%	7%	[95]
*Saccharomyces cerevisiae*	1 (Rlm1, functional MEF2 analog)	61%	11%	7%	[96]

**Fig. 2:**
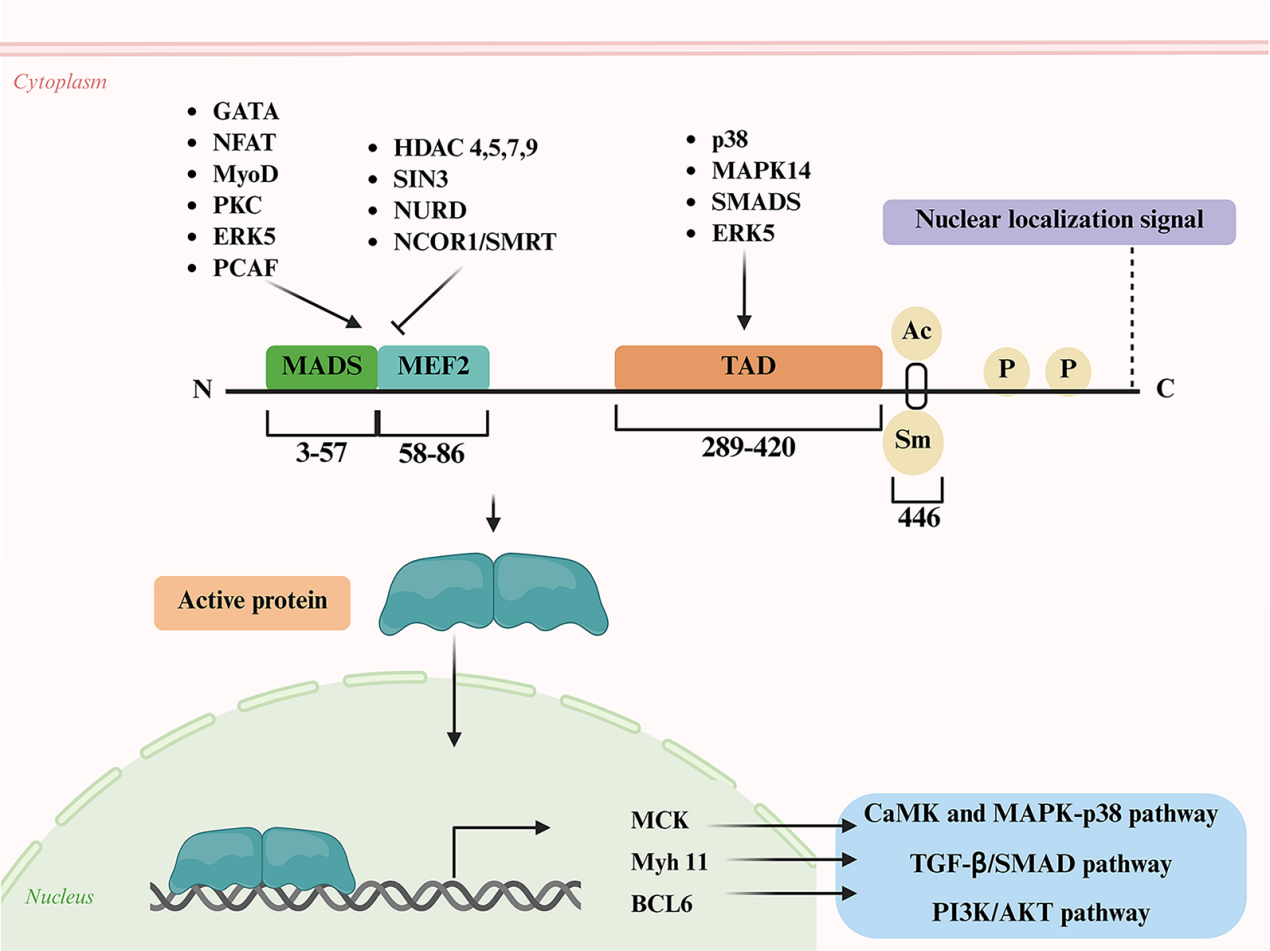
Structure, interacting partners, and regulation of MEF2 protein. The MEF2 protein contains distinct domains that mediate interactions with regulatory proteins and signaling pathways. The N-terminal MADS box (responsible for DNA binding and dimerization) and MEF2 domains (which aids in DNA binding specificity) interact with transcription factors and cofactors such as GATA, NFAT, MyoD, PKC, ERK5, PCAF, HDACs, SIN3, NURD, and NCOR1/SMRT. The transactivation domain (TAD) interacts with p38, MAPK14, SMADs, and ERK5. Post-translational modifications such as acetylation (Ac) (Lys-4 and Lys-403), phosphorylation (P) (Ser-408, Thr-312 and Thr-319), and sumoylation (Sm) (Lys- 403, Lys-391 and Lys-439) further regulate MEF2 activity. Upon activation, MEF2 translocate to the nucleus and regulates the expression of genes such as MCK, Myh11, and BCL6. These targets are influenced by upstream signaling cascades, including the CaMK/MAPK-p38, TGF-β/SMAD, and PI3K/AKT pathways. (Created in BioRender. Kabekkodu, S. (2025) https://BioRender.com/5oa77tb)

Structurally, all MEF2 proteins are members of the MADS-box TF superfamily with a highly conserved MADS domain and a MEF2 domain situated upstream in their N terminal region. These domains are crucial for dimerization, DNA binding and interactions with cofactors. The MEF2 protein recognizes a consensus DNA-binding motif with the sequence (T/C)TA(A/T)4TA(G/A), which is bound by both homodimers and heterodimers formed among MEF2 family members [15, 33]. Their C-terminal transactivation domain (TAD) is more flexible and enables functional diversification by interacting with different signalling pathways [34]. MEF2 is activated by p38, MAPK, and ERK5 signalling molecules by phosphorylating conserved amino acids in the TAD in T cells and fibroblasts [35]. Post-translational regulation maintains MEF2 in a dynamic state to respond to changes in cellular environment during development. The MEF2 family members show tissue specific expression that overlap during different developmental stages (Fig. 2). MEF2A, for example, is highly expressed in the heart, skin and brain, and has essential functions in cardiac muscle, whereas MEF2C has very high expression in the brain and is important for neurodevelopment and synaptic function [36]. The less well- characterized MEF2B and MEF2D are implicated in B-cell development, lymphoid malignancy, and neuronal development, with MEF2D being ubiquitously present in brain and muscle tissues [12]. MEF2 activity is also regulated by post-translational modifications (phosphorylation, sumoylation, and ubiquitination), and alternative splicing. MEF2A, MEF2C, and MEF2D are phosphorylated and activated by BMK1 (big mitogen-activated protein kinase 1), whereas MEF2B is regulated by specific PKA-mediated phosphorylation [37]. Phosphorylation also triggers sumoylation on adjacent residues in MEF2B-D, which normally represses their transcriptional activity. Remarkably, MEF2C possesses a splice variant that lacks the sumoylation site and thus escapes repression by this process. Furthermore, phosphorylation can act as a priming signal for ubiquitination, targeting MEF2 proteins for proteasomal degradation. This post-translational cascade provides a stringent mechanism to rapidly terminate MEF2 activity and ensures tight control over its transcriptional programs [14].

**Fig. 3:**
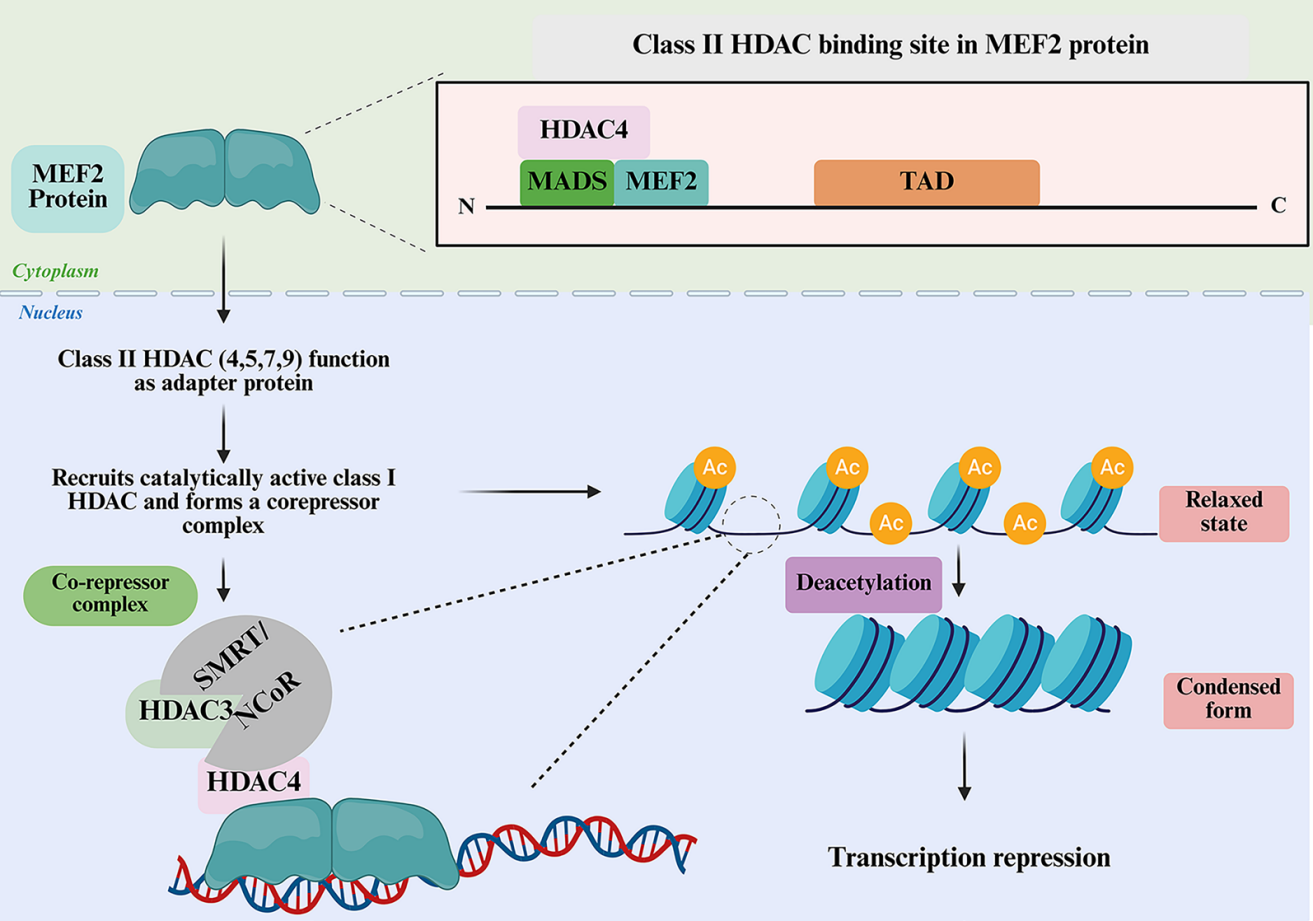
MEF2 transcriptional repression by HDACs. MEF2 proteins localize in the nucleus and contain an N-terminal MADS and MEF2 domain, with a C-terminal transactivation domain (TAD). Class II HDACs (HDAC4, 5, 7, 9) shuttle between the cytoplasm and nucleus and, upon nuclear localization, bind to MEF2 through the N-terminal domain. These Class II HDACs act as adaptor proteins, recruiting catalytically active Class I HDACs (e.g., HDAC3) together with co-repressors such as SMRT and NCoR to form a repression complex on chromatin. The recruited complex deacetylates histones, converting chromatin from a relaxed, transcriptionally active state to a condensed state, thereby repressing gene transcription. (Created in BioRender. Kabekkodu, S. (2025) https://BioRender.com/5oa77tb)

HDACs not only regulate gene expression but also serve as essential binding partners for MEF2 TFs, thereby modulating their transcriptional activity. Class IIa HDACs (HDAC4, HDAC5, HDAC7, and HDAC9) bind directly to the N-terminal MADS/MEF2 domain of MEF2 proteins and repress their transcriptional activities [38]. Although they lack intrinsic deacetylase activity, they function primarily as adaptor proteins that recruit catalytically active class I HDACs, particularly HDAC1 and HDAC3, through interactions mediated by nuclear receptor corepressors such as SMRT (silencing mediator of retinoid and thyroid receptor) and NCoR (Nuclear Receptor Corepressor) [39, 40]. These corepressors form large multiprotein complexes consisting of HDAC1–SIN3, NuRD, and HDAC3–NCoR/SMRT, which stabilize the interaction with MEF2 and enable histone deacetylation at MEF2 target loci, resulting in chromatin compaction and transcriptional repression [41] (Fig. 3). Furthermore, class IIa HDAC activity and intracellular localization are also regulated by phosphorylation. Upon phosphorylation, HDACs get complexed with 14-3-3 protein and are exported from the nucleus to the cytoplasm to facilitate MEF2-dependent gene expression [42]. Lysine acetylation of cytosolic and mitochondrial proteins has been shown to control a vast array of biological functions [43]. Interestingly numerous cancers have reported abnormalities of MEF2-HDAC pathway. For instance, overexpression of class IIa HDAC, especially HDAC9, was correlated with decreased MEF2 activity and poor survival in estrogen receptor-positive (ER+) breast cancers [44]. HDAC9 represses MEF2D function in oral squamous cell carcinoma and supports tumorigenesis [45]. Besides, role of MEF2 and class IIa HDACs is identified in leiomyosarcoma, a soft tissue malignancy [46]. Thus, MEF2 and its interaction with HDACs may play pivotal role in carcinogenesis.

## Mechanistic insights into MEF2 mediated cancer development and progression

**Fig. 4:**
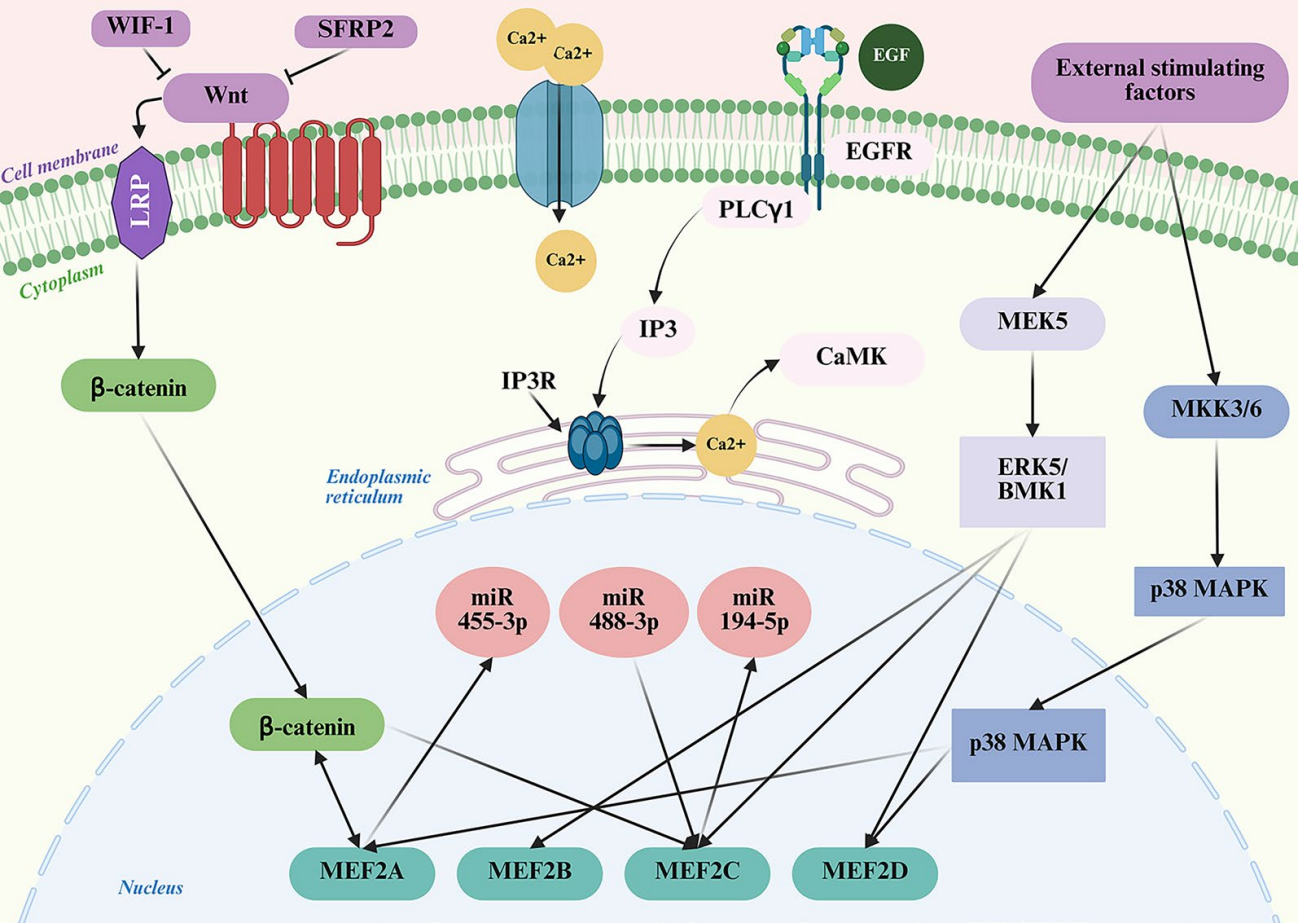
Illustration of signalling pathways interacting with MEF2 and its family members. Multiple signaling pathways converge to regulate MEF2 activity. Wnt signaling activates β-catenin, which cooperates with MEF2 factors in the nucleus. EGF binding to EGFR activates PLCγ1, leading to IP3 production, Ca²⁺ release through IP3R, and CaMK activation, which enhances MEF2 function. External stimulating factors activate MEK5–ERK5 and MKK3/6–p38 MAPK pathways, both of which regulate MEF2 proteins. Post-transcriptional regulation is provided by microRNAs like miR-455-3p, miR-488-3p, and miR-194-5p. These integrated pathways collectively modulate MEF2 isoforms (MEF2A, MEF2B, MEF2C, MEF2D), influencing their activity in gene regulation (Created in BioRender. Kabekkodu, S. (2025) https://BioRender.com/5oa77tb)

The MEF2 proteins serve as switches that regulate fundamental cell processes like proliferation, differentiation, and cell death [47]. MEF2 proteins are regulated by multiple key signalling pathways, including: (A) calcium-dependent signalling, which activates in response to fluctuating calcium concentrations; (B) MAPK pathways, typically stimulated by growth factors and cellular stress; (C) Wnt/β-catenin signalling, which plays pivotal roles in development and tumorigenesis; and (D) the PI3K/AKT pathway, responsible for mediating cell growth and survival. Additionally, MEF2 activity is modulated by various epigenetic mechanisms (Fig. 4).

### Myocyte enhancer factor 2 A (MEF2A)

MEF2A is a TF located on chromosome 15q26.3 and has been classically studied in muscle growth and cardiovascular processes [48]. Recent research has also shown it to play a crucial role in several types of cancer where its function varies according to the cell type and molecular pathways. In gastric cancer (GC), MEF2A plays a role in cisplatin resistance [28]. MEF2A promotes PGC1α transcription, a central molecule linked with mitochondrial biogenesis, and augment mitochondrial activity. At the same time, it represses KEAP1 expression, leading to activation of the NRF2 pathway, which restrains reactive oxygen species (ROS) accumulation. This is responsible for apoptosis resistance and survival of GC cells under chemotherapy stress [49]. In colorectal cancer (CRC), MEF2A acts as an oncogene and promotes tumor growth via activation of epithelial-to-mesenchymal transition (EMT) and Wnt/β-catenin pathway [50]. MEF2A binds directly on ZEB2 and CTNNB1 promoters to upregulate their transcription to promote EMT and Wnt/β-catenin pathway. Induction of ZEB2 promotes EMT to enhance migratory and invasive ability cancer cells. CTNNB1 is a gene encoding β-catenin, the core protein of the Wnt signalling pathway, involved in cell proliferation and survival [50]. Though oncogenic in GC and CRC, MEF2A has tumor-suppressing function in renal cell carcinoma (RCC). MEF2A overexpression in RCC cells suppresses the Wnt/β-catenin signalling pathway, thus reducing downstream target like Cyclin D1, c-Myc, and Survivin. The suppression impairs cell proliferation, migration, invasion, and triggers apoptosis [51]. In acute myeloid leukemia (AML), MEF2A promotes cancer progression by regulating non-coding RNAs that control gene expression. MEF2A is the TF for circPVT1, a circular RNA molecule that functions as a molecular sponge for the tumor suppressor microRNA miR-455-3p. MEF2A suppresses miR-455-3p indirectly by increasing circPVT1 resulting in increased cell growth and survival in AML cells [52]. Recent studies have identified MEF2A as a novel transcriptional activator that promotes pancreatic cancer progression through upregulation of PSMD14 expression. PSMD14 promotes deubiquitination of RBM15B, resulting in enhanced m6A modification-stability of SPON2 mRNA, which, in turn, promotes pancreatic cancer cell proliferation, migration, and invasion [53]. In hepatocellular carcinoma (HCC), MOTS-c-induced activation of MEF2A has been found to counteract hypoxia-caused TRAIL resistance to apoptosis [54]. These findings demonstrate dual role of MEF2A in cancer and raise the possibility that it could be a target for cancer treatment.

### Myocyte enhancer factor 2B (MEF2B)

MEF2B belongs to the MEF2 family of TF, located at 19p13.11 crucial for B-cell development and muscle transformation [55]. Mutations in MEF2B is contributes to germinal center (GC)-type B cell lymphomas, including diffuse large B-cell lymphoma (DLBCL) and follicular lymphoma (FL). Approximately 11% of DLBCL and 12% of FL harbor MEF2B mutations. Mutations in MEF2B enhances its transcription activity to promote BCL6 oncogene to promote lymphomagenesis [56]. In DLBCL, shared somatic MEF2B mutations (mainly within the N-terminal MADS and MEF2 domains) result in loss of negative regulation over MEF2B transcriptional activity. These mutations compromise interaction of MEF2B with transcriptional repressors like class IIa HDACs resulting in constitutive activation of MEF2B. BCL6 overexpression brought on by MEF2B activation promotes proliferation and inhibits B-cell death and differentiation, which in turn promotes lymphomagenesis. Therefore, MEF2B mutations acts as oncogenic drivers in a subset of DLBCL through interference with normal transcriptional repression mechanisms and activation of a key oncogene [37]. Recent studies have revealed that post-translational modifications such as phosphorylation of selected residues have also been shown to control MEF2B activity. For example, phosphorylation at serine-324 residue promotes the recruitment of MEF2B with co-repressors and its degradation by the CUL3/KLHL12 ubiquitin complex, thereby guaranteeing regulated transcriptional activity and protein stability. In B-cell lymphomas somatic mutations at C-terminal portion of MEF2B were discovered. Because these mutations affect phosphorylation at S324, the MEF2B protein is less likely to degrade and is more stable. This could further increase MEF2B transcriptional activity via its interaction with SWI/SNF chromatin remodeling complex to activate anti-apoptotic genes such as BCL6, which enables germinal center expansion and lymphomagenesis [18]. MEF2B contributes to normal germinal center development within the lymph node microenvironment, where it regulates B cell maturation and differentiation. MEF2B downregulation leads to defective GC formation, whereas mutations like D83V promote excessive GC growth and may lead to lymphoma, particularly in conjunction with overexpression of oncogene such as BCL2. Additionally, D83V mutation overcomes MEF2B inhibition by HUCA complex (HIRA/UBN1/CABIN1/ASF1) and class IIa HDACs, resulting in a hyperactive transcriptional state relative to wild-type MEF2B [56]. While MEF2B’s function in B-cell lymphomas is well-established, its role in other cancers is still unclear.

### Myocyte enhancer factor 2 C (MEF2C)

The MEF2C gene maps at the short arm of chromosome 5 at position 5q14.3 of the human genome. The locus has been associated with numerous neurodevelopmental and immunological processes, including the critical role of the gene in disease and development [57]. MEF2C is a TF that is required for the initial process of embryonic development, in which it helps in the development of critical organ systems such as the cardiovascular system, central nervous system (CNS), and the hematopoietic lineages [58]. In the hematopoietic system, MEF2C has a significant function of guiding the differentiation of the lymphoid cells, B-cells and T-cells. In the nervous system, MEF2C is involved in neuron survival, synaptic plasticity, and neurogenesis [59]. Its function is stringently regulated by post-translational modifications including phosphorylation, acetylation, and sumoylation as well as through association with co-activators p300/CBP and co-repressors like class IIa HDACs [59]. In cancer, MEF2C exhibits strongly context-dependent functions. In gliomas, circVCAN acts as a molecular sponge for miR-488-3p, leading to MEF2C upregulation. MEF2C promotes glioma progression by transcriptionally upregulating JAGGED1 expression, thereby enhancing tumor cell proliferation and metastatic potential. Blockage of circVCAN/miR-488-3p/MEF2C JAGGED1 axis suppresses tumor invasion and proliferation suggesting a therapeutic potential for this axis [60]. MEF2C is significantly downregulated (PMID: 23618224) [61] in lung adenocarcinoma (LUAD) and correlates with poor prognosis, decreased overall survival, and immune cell infiltration modification [62]. MEF2C expression has also been linked with sensitivity to chemotherapeutic drugs such as Topotecan, Irinotecan, Panobinostat, and Nilotinib, indicating a function related to treatment response and immune regulation in LUAD [62]. In prostate and breast cancers, MEF2C has been implicated in bone metastasis. Cell invasion and metastasis was correlated with downregulation of tumor-suppressive miR-524-5p and upregulation of MEF2C. Conversely, overexpression of miR-524-5p represses MEF2C and metastatic activity [63]. Likewise, in triple-negative breast cancer (TNBC), MEF2C expression is upregulated whereas miR-194-5p expression is downregulated. Silencing MEF2C or re-expression of miR-194-5p reverses EMT and thereby decreased invasiveness of TNBC cells [64]. MEF2C overexpression is correlated with poor overall survival, more invasive tumor, and greater expression of immunosuppressive factors in gastric cancer, suggesting its regulatory role in the tumor immune microenvironment [65]. MEF2C enhances the malignancy of HCC by enhancing VEGF-induced vasculogenic mimicry and Wnt/β-catenin pathway activation [66]. Also, in lung cancer, MEF2C drives resistance to ferroptosis, a process of iron-dependent cell death [67]. Collectively, these findings establish MEF2C as a context-dependent regulator in cancer biology; its function as either oncogenic or tumor-suppressive depends on tumor type and cellular microenvironment. By regulating EMT programming, immune modulation, metastatic progression, and therapy resistance, MEF2C emerges as both a promising prognostic biomarker and therapeutic target.

### Myocyte enhancer factor 2D (MEF2D)

MEF2D located at chromosome 1 position at 1q12-q23 is a TF that responds dynamically to intracellular signals like calcium influx, MAPK signalling, and stress stimuli as a transcription switch to control gene expression [68]. Depending on the tissue context, MEF2D can function as either an oncogene or a tumor suppressor, revealing its complex role in cancer biology. It facilitates tumorigenesis by driving cell cycle progress, migration, and chemoresistance [69]. MEF2D knockout models have revealed that the loss of MEF2D disrupts leukemia growth as well as induction of myeloid differentiation [70]. CEBPE is a myeloid differentiation factor transcriptional activator important for differentiation. MEF2D represses CEBPE to block differentiation to sustain the leukemic condition [71]. HOXA9 positively regulates MEF2D expression, and its downregulation accounts for DOT1L inhibitor antileukemic activity [71]. In B-cell acute lymphoblastic leukemia (B-ALL), rearrangements in MEF2D are found in about 2.4–5.3% of cases and show adverse prognosis. These fusions include the N-terminus of MEF2D with genes like BCL9 and HNRNPUL1. The resulting fusion proteins are augmented in function and stable over wild-type MEF2D, resulting in aberrant transcriptional programs. MEF2D fusion patients present with early relapses and a median overall survival of approximately 11 months [72]. In hepatocellular carcinoma (HCC), MEF2D promotes intrahepatic metastasis by perceiving pro-metastatic niche cues. It transactivates integrins ITGB1 and ITGB4 to facilitate early seeding and late colonization of disseminated cancer cells. An integrin-FAK circuit regulates MEF2D stability through a phosphorylation-dependent deubiquitination switch to escape MDM2-mediated degradation. Clinically, the USP14(pS432)-MEF2D-ITGB1/4 feedback loop is hyperactivated and associated with poor prognosis [73]. In contrast to its oncogenic function in HCC, MEF2D acts as a tumor suppressor in breast cancer [69]. Its knockout in mammary epithelial cells causes neoplastic transformation, i.e., increased growth, loss of contact inhibition, and anchorage-independent growth. Mechanistically, MEF2D loss causes EMT and oncogenic signalling pathways such as AKT, ERK, and Hippo-YAP. Decreased expression of MEF2D is seen in TNBC and is associated with poorer overall and relapse-free survival [69]. Emerging evidence implicates MEF2D in diverse malignancies, spanning hematological cancers to solid tumors (including lungs, pancreatic, and colorectal carcinomas). This broad involvement with various cancers has positioned MEF2D as an important candidate for further investigation, with potential applications such as: (A) a diagnostic marker, (B) a prognostic indicator, (C) a predictor of treatment response, and (D) a novel therapeutic target.

Role of MEF2 family proteins in different types of human cancer (Table 2).

**Table 2:** Role of MEF2 family proteins in different types of human cancer

Cancer type	MEF2 protein type	Role of MEF2	Gene interaction and mechanism	Signalling pathway	Reference
Gastric Cancer (GC)	MEF2A	Enhances cisplatin resistance by promoting mitochondrial biogenesis	Transcriptional activation of PGC1α and inhibition of KEAP1, leading to increased mitochondrial function and reduced ROS levels	KEAP1/NRF2 signalling	[28]
Gastric Cancer (GC)	MEF2A	Mitigates cisplatin resistance by activating NFKBIA, leading to NF-κB pathway inhibition	Transcriptional activation of NFKBIA, resulting in decreased phosphorylation of p65 and cytoplasmic retention	NF-κB signalling	[49]
Hepatocellular Carcinoma (HCC)	MEF2D	Enhances sorafenib resistance by inhibiting ferroptosis	Upregulation of ACSL3 expression, leading to reduced lipid peroxidation and ferroptosis	Ferroptosis pathway	[97]
Hepatocellular Carcinoma (HCC)	MEF2D	Promotes intrahepatic metastasis by stabilizing MEF2D protein levels	Inhibited polyubiquitination via integrin-FAK feedback loop; MDM2-mediated degradation suppressed	Integrin-FAK signalling	[98]
Pancreatic Cancer (PC)	MEF2A, MEF2C, MEF2D	Potential biomarkers; associated with tumor progression	Direct regulation of FNIP1 and FNIP2, sustaining mTORC1 activation and tumor progression	mTORC1 signalling	[99]
Acute Myeloid Leukemia (AML)	MEF2A	Contributes to malignancy by regulating circular RNA	Transcriptional activation of circPVT1, promoting leukemogenesis	MEF2A/circPVT1/miR-455-3p/MCL1 molecular axis	[52]
Gastric Cancer (GC)	MEF2D	Facilitates liver metastasis under cytokine stimulation	Induces H1X expression under IL-13 stimulation, promoting metastasis	IL-13-mediated signalling	[100]
Colorectal Cancer (CRC)	MEF2A	Enhances tumor progression by upregulating EMT-related genes	Transcriptional activation of ZEB2 and CTNNB1 (β-catenin), leading to EMT and increased proliferation	Wnt/β-catenin signalling	[50]
Acute Myeloid Leukemia (AML)	MEF2D	Maintains leukemia by repressing differentiation gene CEBPE	Binds to CEBPE enhancer, maintaining repressive chromatin status and inhibiting gene transcription	Chromatin remodelling	[71]
Non-Small Cell Lung Cancer (NSCLC)	MEF2D	Targeted by miR-1244 to enhance cisplatin sensitivity	miR-1244 suppresses MEF2D, leading to increased p53 and Bax expression, promoting apoptosis	Cyclin D1-p53 signalling	[101]
Hepatocellular Carcinoma (HCC)		Promotes epithelial-mesenchymal transition (EMT) and invasiveness	Autoregulation of TGF-β1 expression, leading to sustained EMT and increased invasiveness	TGF-β signalling	[83]
Diffuse Large B-Cell Lymphoma (DLBCL)	MEF2B	Mutations lead to deregulated expression of oncogene BCL6	Mutations disrupt interaction with corepressor CABIN1, enhancing BCL6 transcriptional activity	BCL6 signalling	[37]

## Interactions between the MEF2 family and oncogenic pathways

MEF2 proteins function as hub that integrates diverse intracellular signalling pathways and epigenetic regulatory networks to control gene expression regulation. MEF2 proteins activity is regulated by post-translational modifications like phosphorylation, acetylation, and SUMOylation, in addition to interactions with chromatin remodelers, co-repressors, and co-activators [74]. In recent years, evidence has suggested that MEF2 family members play a key role as regulators and effectors for a range of oncogenic signalling pathways and are vital to control EMT, angiogenesis, immune evasion, metabolic reprogramming, and apoptosis resistance. The subsections below explain the interaction between MEF2 family of proteins with oncogenic pathways during carcinogenesis.

### Cyclic AMP-dependent protein kinase A (PKA)

The interaction between PKA and MEF2 is both tissue-specific and context-dependent. For example, activation of the cAMP/PKA pathway phosphorylates MEF2 to enhance its activity and facilitate neuronal survival in cerebellar and cortical granule neurons [75]. Activation of this pathway results in PKA phosphorylation of MEF2 at threonine 20 (Thr-20) increasing the DNA-binding activity of MEF2 [76]. cAMP-PKA pathway is also involved in controlling many essential cellular functions and has been found to contribute to carcinogenesis in certain cancers. Aberrant PKA activation can cause hyper-phosphorylation of MEF2 to enhance its activity as well as gene expression promoting the growth and survival of cancer cells [77]. On the contrary, targeting this pathway inhibited tumor formation by reducing MEF2 activity. Thus, targeting the PKA and MEF2 interaction may be an attractive anti-cancer approach in certain cancers.

### Notch signalling pathway

The Notch signalling pathway and MEF2 functional interplay are critical determinants of cell fate and maintenance hematopoietic lineage balance. Disruption of this interaction is suggested as critical mechanism that promotes leukemogenesis [78]. MEF2C and Notch signalling have opposite functions in lymphoid cell development with critical implications in cancer development [79]. Notch signalling is important for T cell commitment during thymocyte development [80]. For instance, in a study, MEF2C inhibited Notch-dependent T cell differentiation and reprograms the precursor cells into a B-lineage in early thymocyte progenitor acute lymphoblastic leukemia (ETP-ALL). MEF2C does this through activating TFs such as RUNX1 and GATA3, enhancement of interleukin-7 receptor (IL-7R) signalling, and cell survival by overexpression of BCL2. This results in the generation of biphenotypic leukemias that express both T and B cell markers, as in mixed phenotype acute leukemia. Markedly, pharmacologic inhibition of MEF2C function using dasatinib was able to overcome developmental arrest and make leukemic cells steroid sensitive. Therefore, MEF2C represents a promising therapeutic target in cancers that show its aberrant expression [81]. Another leading example is MEF2 TF that plays a pivotal role in maintaining endothelial function and vascular health. Specifically, MEF2 regulates the expression of genes like KLF2 and KLF4, which are crucial for anti-inflammatory and anti-thrombotic responses in endothelial cells. Additionally, MEF2 interaction with Notch signalling pathway, further contributing to vascular homeostasis. Notably, MEF2 deficiency leads to upregulation of TAZ, a coactivator associated with pro-inflammatory and proliferative responses, indicating a shift towards a pro-atherogenic state [17].

### TGF-β signaling

Studies have shown interaction between MEF2 and TGF-β [82]. TGF-β is known for its role as both tumor suppressor and tumor promoter. The TGF-β and MEF2 interaction has been shown to affect EMT processes, a key process connected with cancer metastasis. A study by W. Yu et al. 2014, showed that MEF2 promotes the expression of TGF-β1, establishing a feedback mechanism that enhances TGF-β1 signalling. This signalling cascade mediates phosphorylation-dependent activation of downstream effectors, notably Smad proteins, which amplify EMT-TFs (Snail, Slug, ZEB1). Subsequent repression of E-cadherin and upregulation of mesenchymal markers orchestrate EMT progression. Thus, HCC cells develop increased migratory and invasive potential responsible for tumor metastasis. This study established the existence of TGF-β1 and MEF2 interaction in activation of EMT to promote invasiveness in HCC [83].

**Fig. 5:**
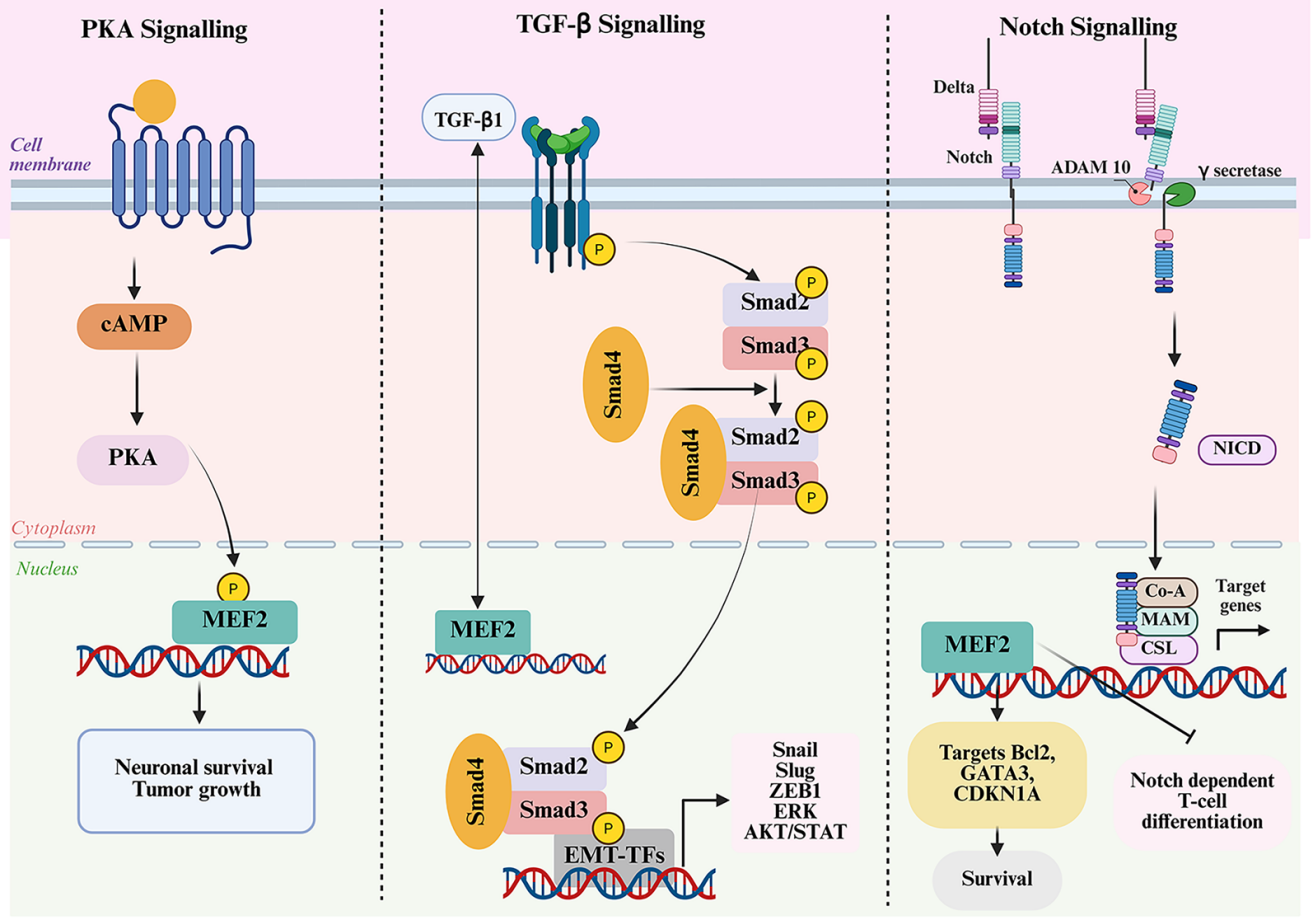
Crosstalk between TGF-β, PKA, and Notch signaling pathways modulating MEF2 transcriptional activity. The cAMP/PKA pathway phosphorylates MEF2, enhancing its transcriptional activity to promote neuronal survival and tumor growth. In the TGF-β pathway, activated receptors phosphorylate Smad2/3, which form complexes with Smad4. These complexes cooperate with MEF2 to activate EMT-related transcription factors (Snail, Slug, ZEB1) and downstream effectors (ERK, AKT/STAT), driving epithelial–mesenchymal transition and metastasis. In the Notch pathway, ligand binding and proteolytic cleavage release the Notch intracellular domain (NICD), which interacts with cofactors (CSL, MAM) and MEF2C. This interaction upregulates survival genes (Bcl2, GATA3, CDKN1A) while inhibiting Notch-dependent T-cell differentiation, contributing to leukemogenesis. The three routes merge to modulate the transcriptional function of MEF2 proteins to regulate various cellular responses such as differentiation, proliferation, and survival (Created in BioRender. Kabekkodu, S. (2025) https://BioRender.com/5oa77tb)

The interaction of PKA, TGF-β, and Notch signalling pathways in controlling MEF2 transcriptional function is illustrated in Fig. 5.

### VEGF mediated angiogenesis

The VEGF-MEF2 axis, essential for physiological vascular development, is aberrantly activated in tumors to promote pathological angiogenesis [84]. VEGF is a strong pro-angiogenic factor with known function as inducer of proliferation, migration, and new vessel formation of endothelial cells. VEGF activation triggers downstream signalling via MAPK and Ca²⁺/calmodulin-dependent pathways, inducing MEF2A and MEF2C TFs [85]. These factors subsequently regulate gene expression programs critical for endothelial sprouting and vascular remodeling. VEGF-induced MEF2 activation was shown to activate pro-angiogenic genes such as Dll4 and Notch pathway members, which in turn maintain endothelial cell fate and vessel branching [84]. The VEGF-MEF2 axis has emerged as a critical mediator of tumor microenvironment remodeling and cancer progression, representing an attractive target for anti-angiogenic therapies in vascularized tumors.

### Epigenetic regulation and stemness

Epigenetics refer to heritable modifications in gene expression that occur without alterations to the underlying DNA sequence [86]. Abnormal epigenetic changes have been implicated in the development and progression of numerous diseases including cancer [87]. Recent studies have suggested that MEF2 activity is carefully regulated by epigenetic mechanisms including histone modifications, chromatin remodeling, and non-coding RNA interactions [44]. These epigenetic regulators not only regulate the expression of MEF2 target genes but also direct stem and progenitor cells to maintain their identity or commitment to a particular lineage. MEF2A and MEF2C, which are expressed to a high degree in the nervous system, are involved in neuronal differentiation, survival, and synaptic plasticity. Genome-wide studies have shown that these TFs bind preferentially to enhancer elements adjacent to neuron plasticity and calcium signalling genes and regulate epigenomic programs required for neuronal function. Their function is regulated by interactions with co-activators such as p300 and CBP, and co-repressors such as class II histone deacetylases (HDACs), suggesting a complex regulatory process that determines gene expression upon receipt of external signals [88]. MEF2C is an important transcriptional regulator in maintaining the balance between stemness and lineage commitment. MEF2C enhances the transcriptional activity of EBF1 (Early B-cell Factor 1) and PAX5 (Paired box-5), two critical regulators of B-cell identity maintenance. In MEF2C-deficient conditions, expression levels of these factors are significantly reduced compromising development at the pro-B to pre-B cell transition stage. MEF2C also regulates targets like IL-7 receptor (IL-7R), an early B-cell growth factor important for survival. Loss of MEF2C leads to abnormal or late B-cell development [89]. Recent studies have identified two novel circular RNAs, circMEF2A1 and circMEF2A2, derived from the MEF2A gene - a well-characterized regulator of muscle development. Both circMEF2A1 and circMEF2A2 are highly abundant in skeletal muscle, where they play a critical role in promoting the differentiation of muscle stem cells (satellite cells) into fully mature myofibers. circMEF2A1 suppresses miR-30a-3p, a microRNA that otherwise suppresses expression of PPP3CA. circMEF2A1 suppression of miR-30a-3p results in higher PPP3CA levels. PPP3CA then stimulates NFATC1, a TF for muscle cell differentiation. circMEF2A2 functions as a molecular sponge for miR-148a-5p, which normally represses SLIT3 expression. By sequestering miR-148a-5p, circMEF2A2 elevates SLIT3 levels, thereby activating the ROBO2/β-catenin signalling pathway - a critical regulator of myogenesis and muscle hypertrophy [90].

## Clinical implication of MEF2 family in cancer

The MEF2 TF family showed both oncogenic and tumor-suppressive roles in cancer, with context-dependent functions that vary depending on cell and cancer type. MEF2 family members are abnormally expressed across a range of cancers, making them valuable candidates for use as molecular biomarkers. Dysregulation of MEF2C is most commonly involved in hematopoietic malignancies, including leukemia and lymphomas. There are, however, studies to suggest that MEF2A and MEF2D, although of special interest in solid tumor (breast carcinoma, hepatocellular carcinoma, and glioblastoma), are also involved in leukemogenesis. Towards this, few studies have targeted MEF2 members using RNA interference (RNAi)-mediated gene silencing approach. In ovarian cancer, for instance, frequent MEF2D overexpression contributes to chemoresistance, making it an attractive target for RNAi-based approaches. Interestingly, MEF2D-targeting using siRNA significantly inhibited tumor progression and enhanced cisplatin chemosensitivity. This RNAi-based nanotherapeutic approach offers a precision strategy to overcome drug resistance in ovarian cancer [91]. An alternative therapeutic strategy involves disrupting MEF2’s interaction with class IIa HDACs - a key regulatory mechanism controlling MEF2-dependent gene expression. The selective HDAC inhibitor MC1568 not only modulates MEF2 activity but also reactivates epigenetically silenced tumor suppressor genes, offering potential for epigenetic reprogramming in cancer treatment [92]. Another study demonstrated that proto-oncogenic kinase SRC phosphorylates MEF2D to increases its transcriptional activity and augmenting MTORC1 signaling. Increase of MEF2D and p-MEF2D levels correlate with more MTORC1 activity in pancreatic cancer tissues and thus SRC-MEF2D-MTORC1 pathway targeting may be a novel treatment approach in pancreatic cancer [93]. The MEF2 family of TF governs many hallmarks of cancer including cellular plasticity, stemness maintenance, and immune evasion, positioning them as potential targets for precision oncology. Therapeutic modulation of MEF2-regulated pathways through direct targeting or via associated non-coding RNAs (e.g., circMEF2A1/2) represents a promising strategy to overcome treatment resistance and improve clinical outcomes in certain cancers [52]. Understanding the diverse mechanisms by which MEF2 drives cancer provides a compelling foundation for the development of novel, mechanism-driven therapeutics.

## Future direction and potential challenges

A critical direction for future research is the systematic elucidation of isoform-specific functions for MEF2A, MEF2B, MEF2C, and MEF2D across diverse malignancies. This should specifically investigate their complex crosstalk with epigenetic machinery, non-coding RNAs (particularly circular RNAs such as circMEF2A), and pivotal signaling cascades that dictate malignant progression. Cutting-edge technologies such as single-cell RNA sequencing, CRISPR screens, and spatial transcriptomics will be key for decoding cell-type-specific functions of MEF2 members in health and diseases. Significant opportunities exist in developing MEF2-targeted diagnostics, particularly through non-coding RNA biomarkers (e.g., circMEF2A and MEF2-regulating miRNAs) for early detection and treatment monitoring. Epigenetic drugs or RNA-based approaches that precisely regulate MEF2 activity may be attempted as novel therapy against cancers that show abnormal expression of these genes. However, major challenges must be addressed, including the complex regulation of MEF2s by post-translational modifications and cofactors, the need for tissue-specific targeting to avoid non-specific effects, and technical challenges connected with in vivo response. Overcoming these difficulties will require innovative approaches to control MEF2’s expression and biological complexity while developing safe, effective precision therapies that fully exploit its therapeutic potential in oncology.

## Data Availability

No datasets were generated or analysed during the current study.

## Abbreviations

MEF2: Myocyte enhancer factor 2
TF: Transcription Factor
MADS: MCM1, agamous, defeciens, serum response factor
PTM: Post-translational modifications
HDAC: Histone deacetylase
MAPK: Mitogen-activated protein kinase
STAT3: Signal transducer and activator of transcription 3
MCK: Muscle creatine kinase
ERK: Extracellular signal-regulated kinase
TAD: Transactivation domain
TGF-β1: Transforming growth factor beta 1
EMT: Epithelial-mesenchymal transition
NURD: Nucleosome remodelling and deacetylase
SIN3: Switch-independent 3
NCOR1: Nuclear receptor corepressor 1
VEGF: Vascular endothelial growth factor
PI3K/AKT: Phosphatidylinositol 3-kinase/Protein kinase B
NRF2: Nuclear factor erythroid 2-related factor 2
KEAP1: Kelch-like ECH-associated protein 1
CDK: Cyclin-dependent kinase
EGFR: Epidermal growth factor receptor
CDKN1A/p21: Cyclin-dependent kinase inhibitor p21
BMK1: Big mitogen-activated protein kinase 1
FOXM1: Forkhead box protein M1
ZEB2: Zinc finger E-box binding homeobox 2
CTNNB1: Catenin beta 1
circPVT1: Circular RNA derived from the PVT1 gene
PSMD14: Proteasome 26 S subunit non-ATPase 14
CUL3: Cullin 3
KLHL12: Kelch like 12
SWI/SNF: SWItch/Sucrose non-fermentable
RBM15B: RNA binding Motif protein 15B
SPON2: Spondin-2
TRAIL2: Tumor necrosis factor-related apoptosis-inducing ligand
HIRA/UBN1/CABIN1/ASF1: HIRA, Ubinuclein-1 (UBN1), Calcineurin-binding protein 1 (CABIN1), and histone chaperone ASF1a
JAGGED1: Jagged canonical Notch ligand 1
HOXA9: Homeobox A9
CEBPE: CCAAT enhancer-binding protein epsilon
DOT1L: Disruptor of telomeric silencing 1-like
BCL9: B-cell CLL/lymphoma 9
HNRNPUL1: Heterogeneous nuclear ribonucleoprotein U-like 1
FAK: Focal adhesion kinase
ITGB: Integrin subunit beta
USP14: Ubiquitin-specific protease 14
YAP: Yes-associated protein
RUNX1: Runt-related transcription factor 1
GATA3: GATA binding protein 3
KLF: Kruppel-like factor
TAZ: Transcriptional co-activator with PDZ binding motif
PAX-5: Paired box gene 5
NFATC1: Nuclear factor of activated T cells1
SLIT3: Slit Guidance Ligand 3
MTORC1: Mechanistic target of rapamycin kinase complex 1
RENCA: Renal Cell Carcinoma, AML- Acute myeloid leukemia
TNBC: Triple-negative breast cancer
NSCLC: Non-small cell lung cancer
CRC: Colorectal cancer
RCC: Renal cell carcinoma
HCC: Hepatocellular carcinoma
siRNA: Small Interfering RNA
ROS: Reactive oxygen species
miR: Micro RNA
CRISPR: Clustered regularly interspaced short palindromic repeats
